# Supplementary material for: Magmatic surge requires two-stage model for the Laramide orogeny
Source: Nat Commun. 2023 Jun 29;14:3841. doi: 10.1038/s41467-023-39473-7 (PMC10310782; doi:10.1038/s41467-023-39473-7)
Supplement: Supplementary file 1 — Supplementary Information [file 41467_2023_39473_MOESM1_ESM.pdf]

Magmatic Surge Requires Two-Stage Model for the Laramide Orogeny

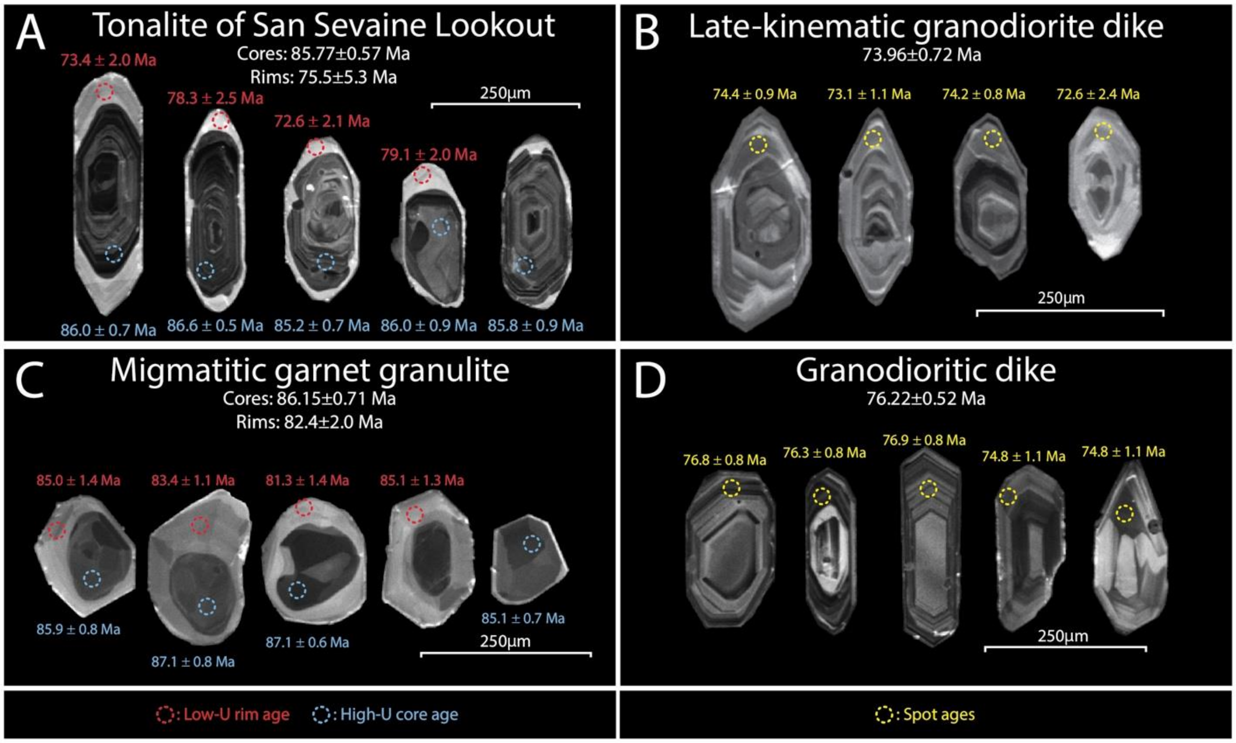

Supplementary Figure 1

Supplementary Figure 1. Selected SEM-cathodoluminescence (CL) images of plutonic zircons dated in this study. SEM-cathodoluminescence images of igneous and metamorphic zircons from tonalite and granodiorites in the eastern San Gabriel Mountains. Uncertainties on dates are 1SD from SHRIMP-RG. Tera-Wasserburg and weighted average age plots are shown in Supplementary Figure 2A-I.

San Gabriel Mountains (San Gabriel block)

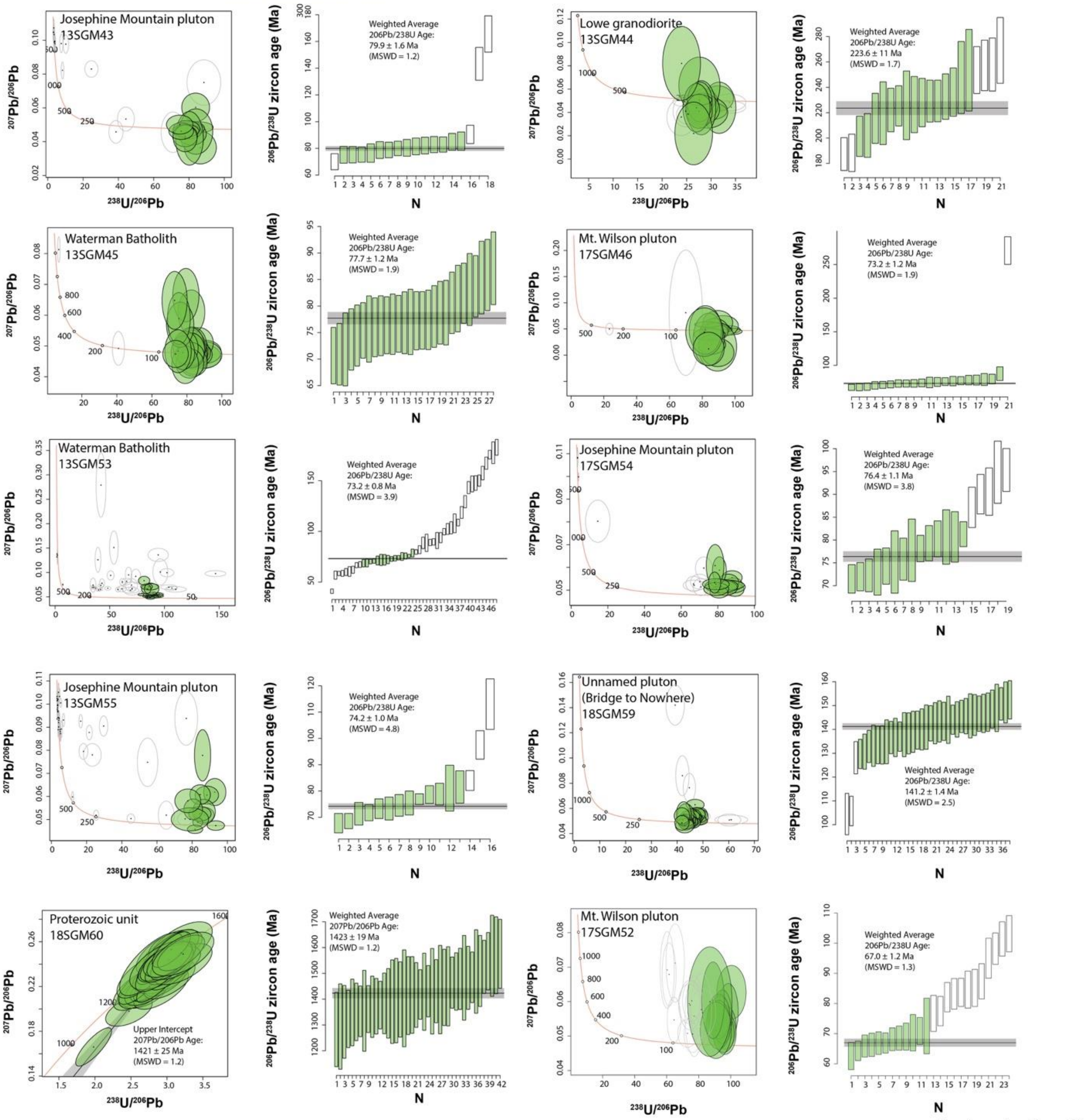

Supplementary Figure 2A

San Gabriel Mountains (Cucamonga terrane)

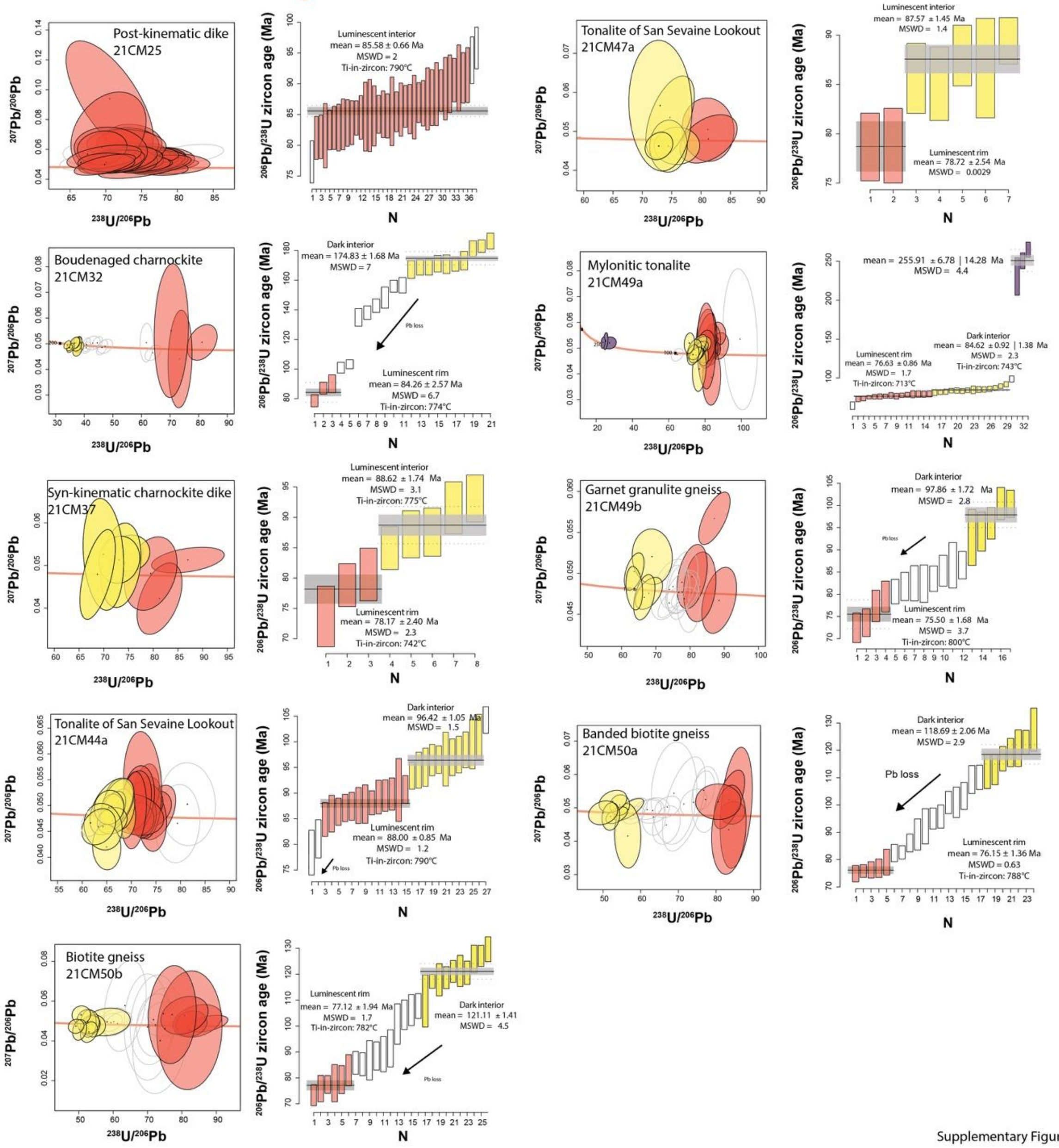

Supplementary Figure 2B

San Gabriel Mountains (Cucamonga terrane)

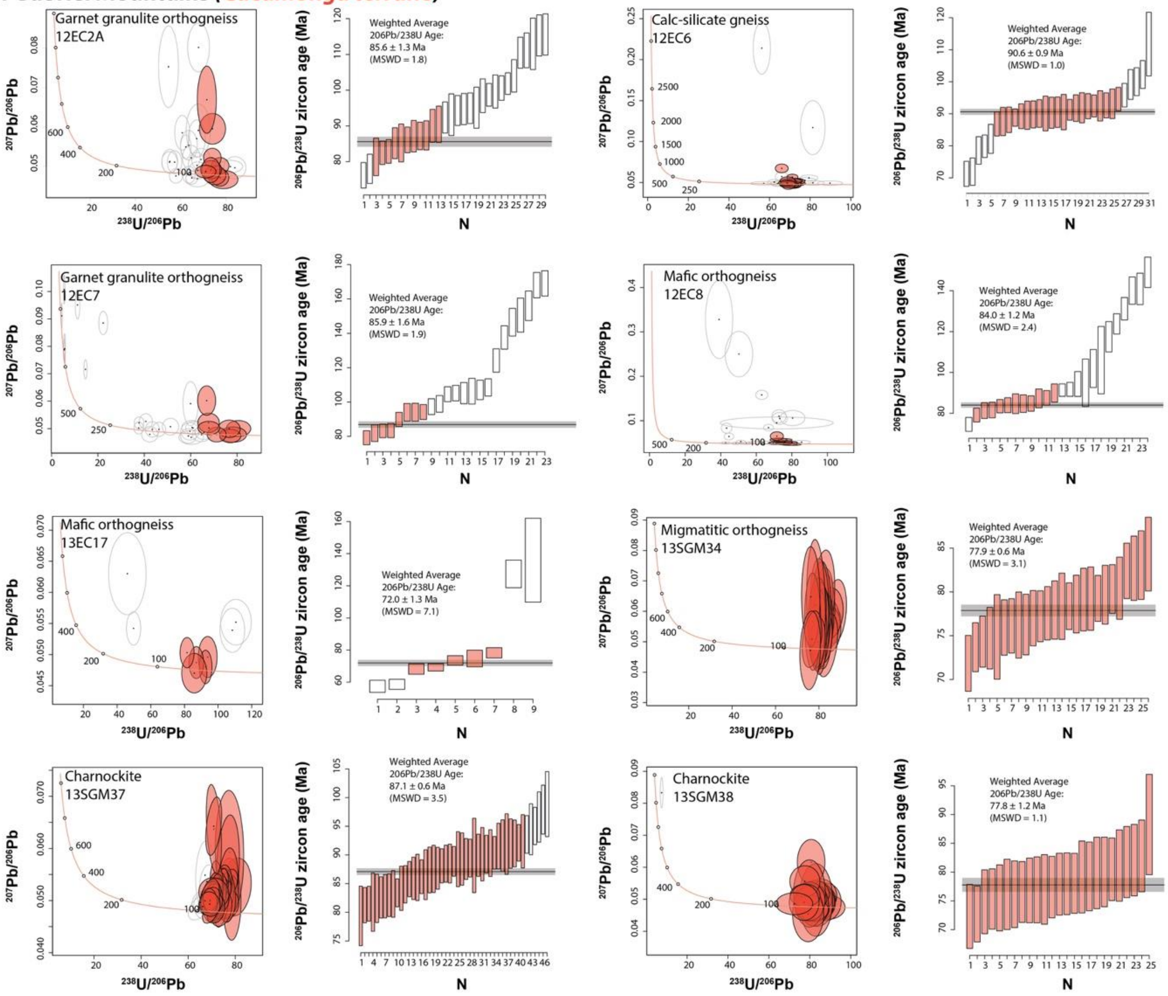

Supplementary Figure 2C

San Gabriel Mountains (Cucamonga terrane & San Gabriel block)

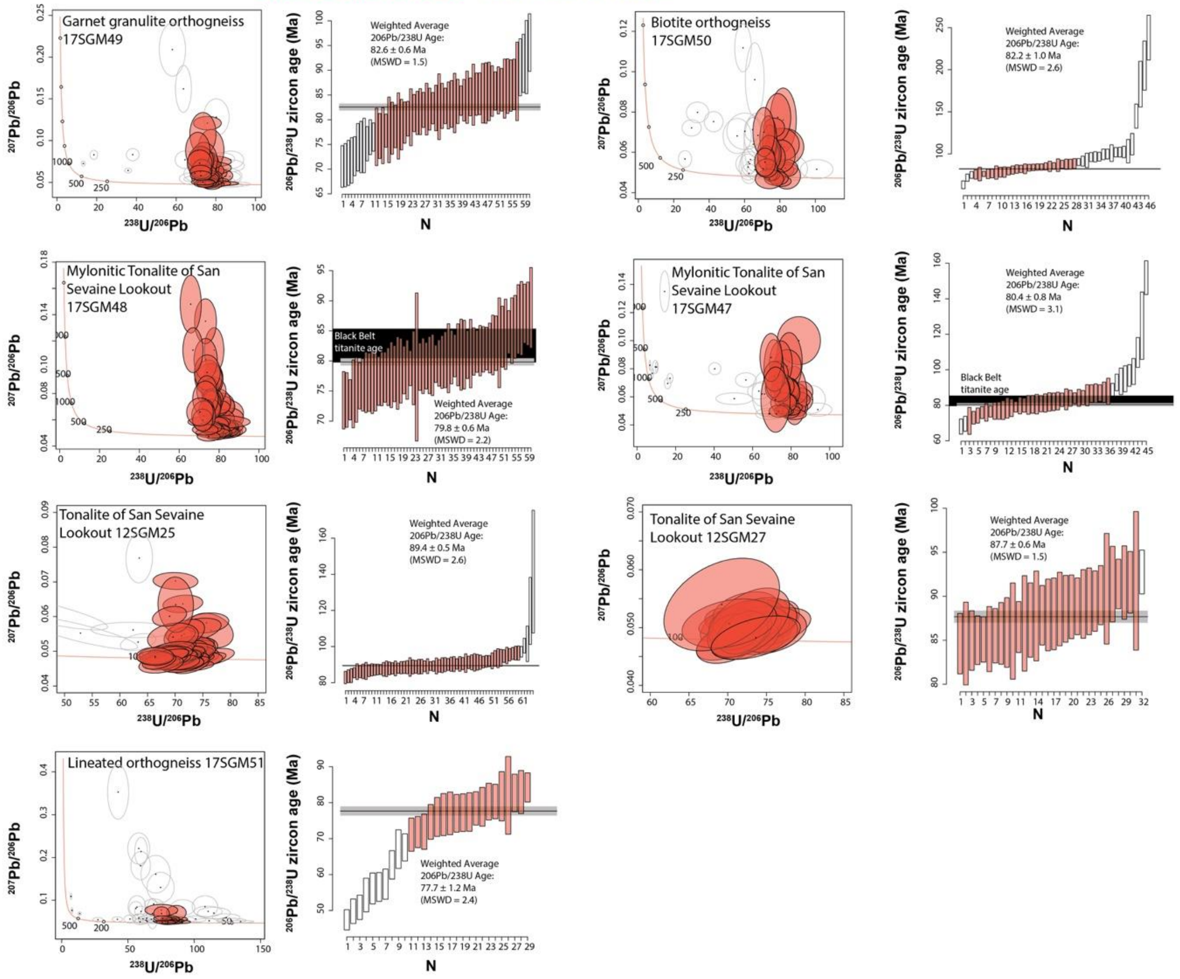

Supplementary Figure 2D

Alamo Mountain block

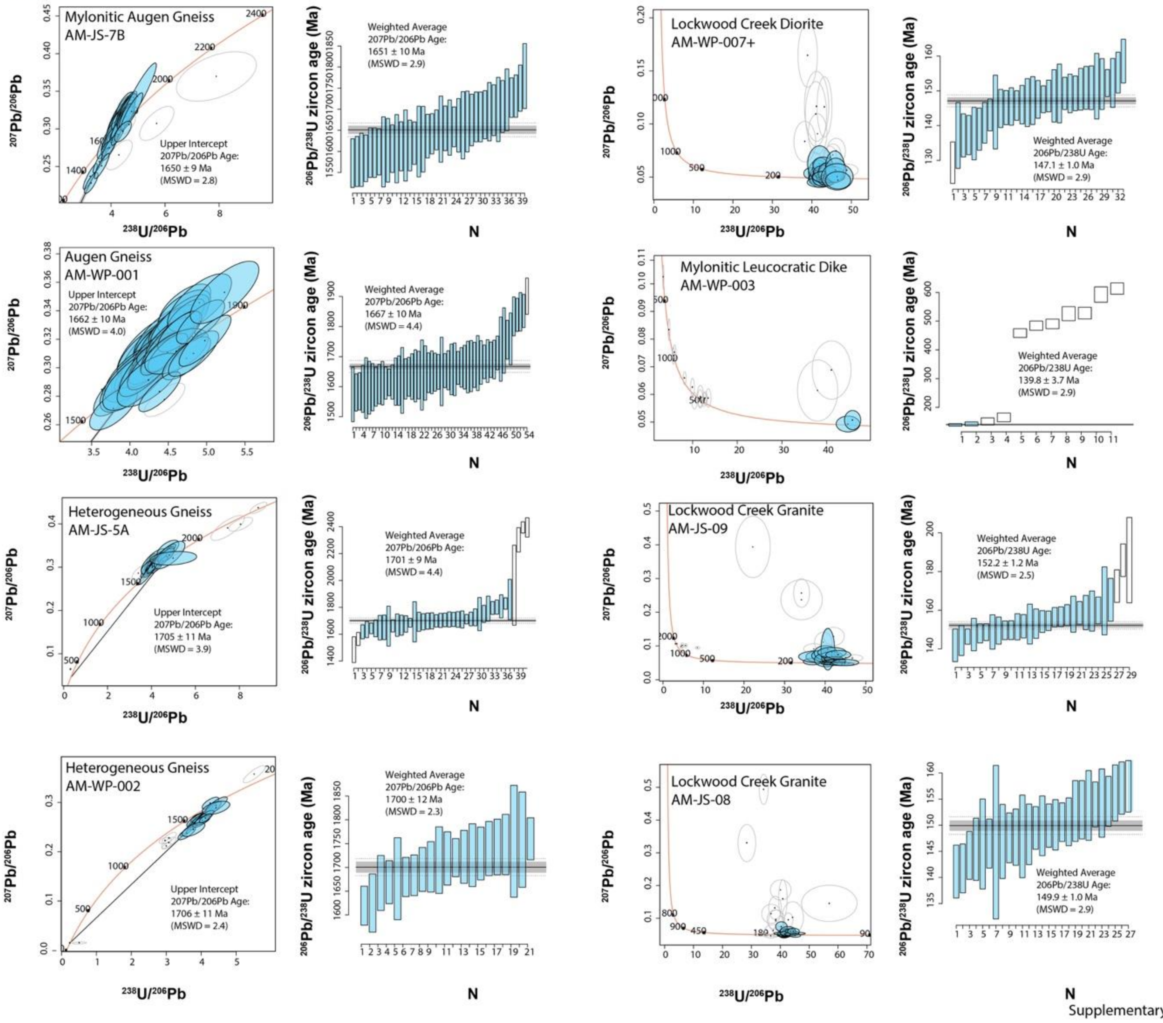

Supplementary Figure 2E

Alamo Mountain block

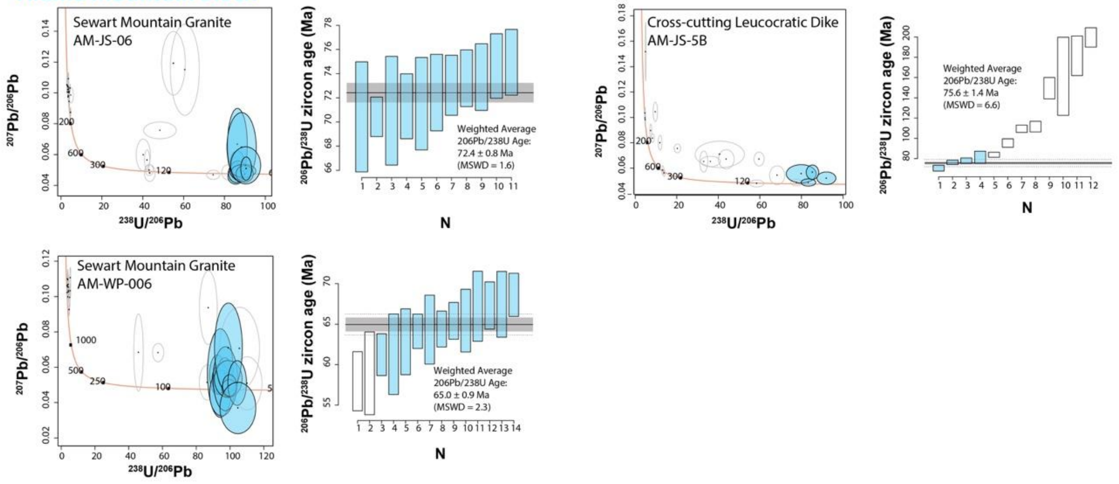

Supplementary Figure 2F

Pine Mountain block

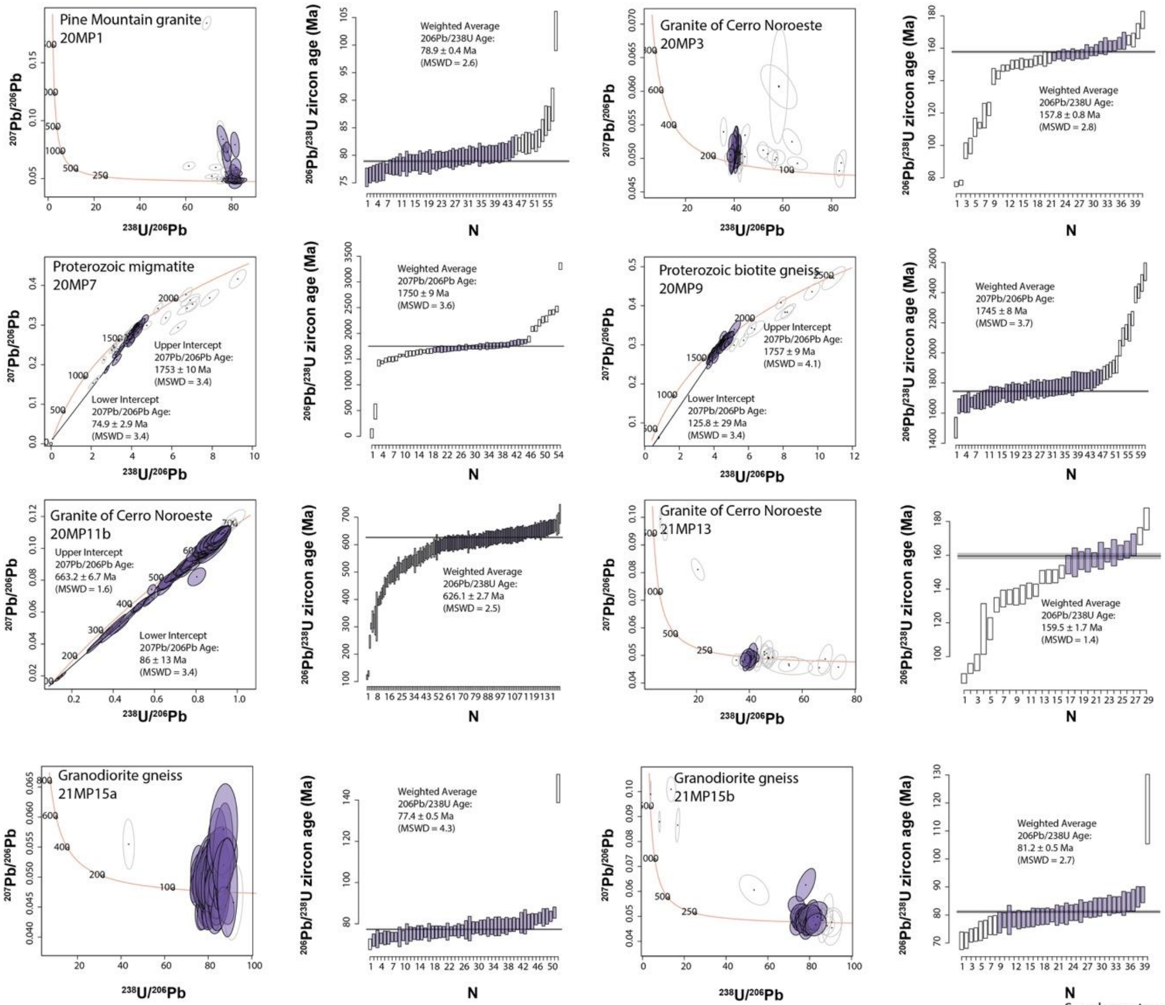

Supplementary Figure 2G

Pine Mountain block

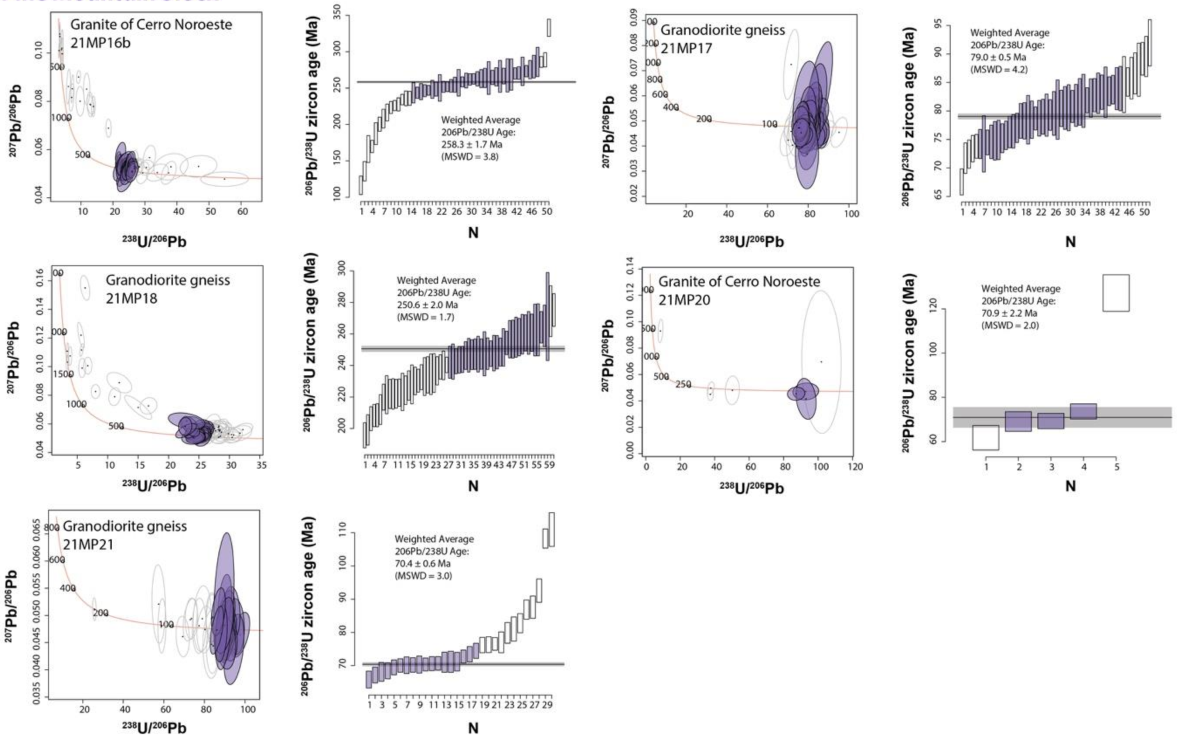

Supplementary Figure 2H

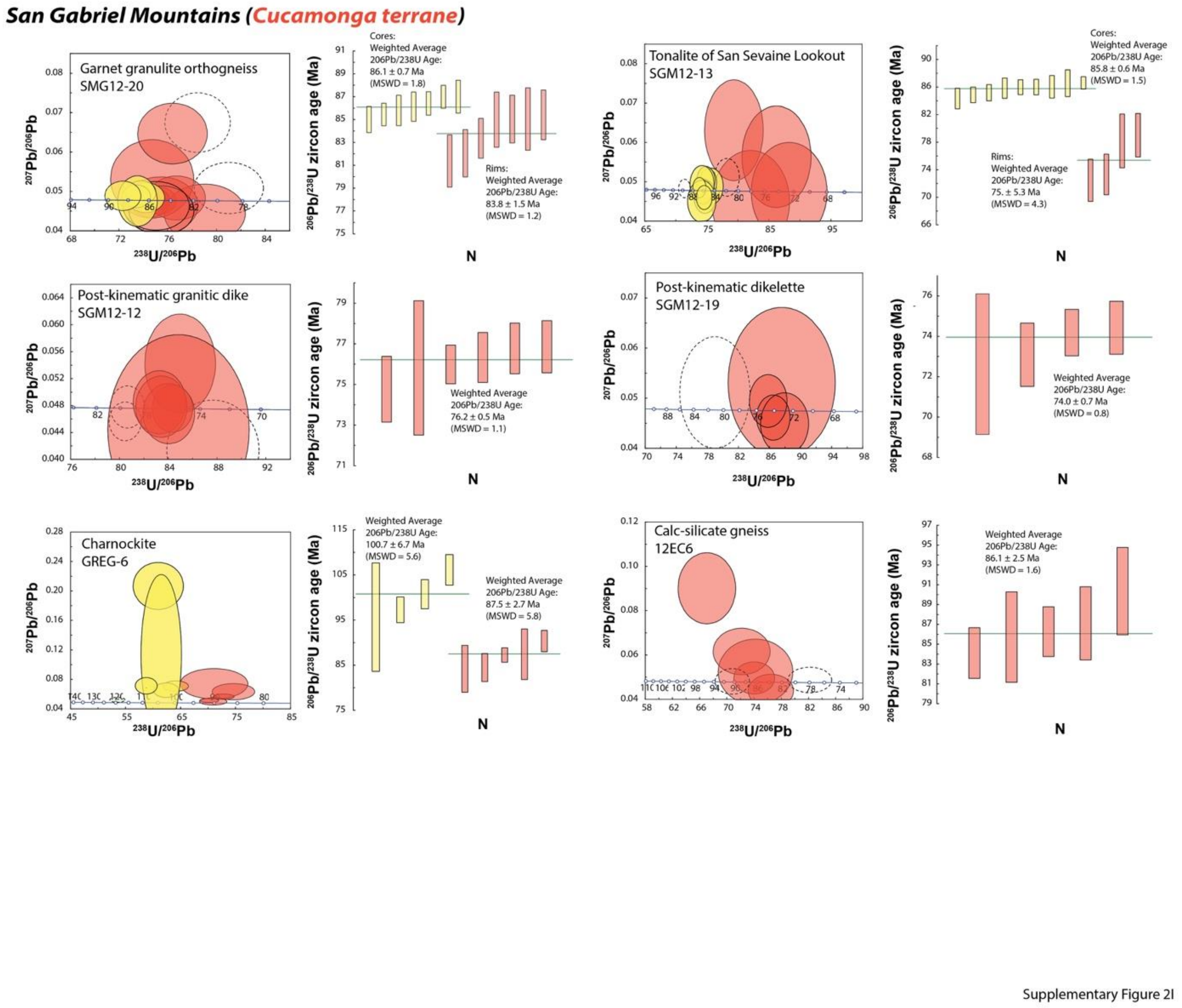

Supplementary Figure 2A-I. Weighted average plots and Tera-Wasserburg diagrams for plutonic samples dated in this study. Dates used in calculations are shown by colored error ellipses, and rejected samples shown in grey. Error ellipses show 2σ total uncertainty for individual spot analyses, and arrows indicate possible lead loss. Data in Supplementary Figure 2A-H are LA-SF-ICPMS data collected at California State University Northridge, whereas data shown in Supplementary Figure 2I are from the Stanford-USGS SUMAC (SHRIMP-RG) laboratory. U-Pb isotope data were plotted using IsoplotR (Vermeesch, 2018). MSWD = Mean square of weighted deviates.

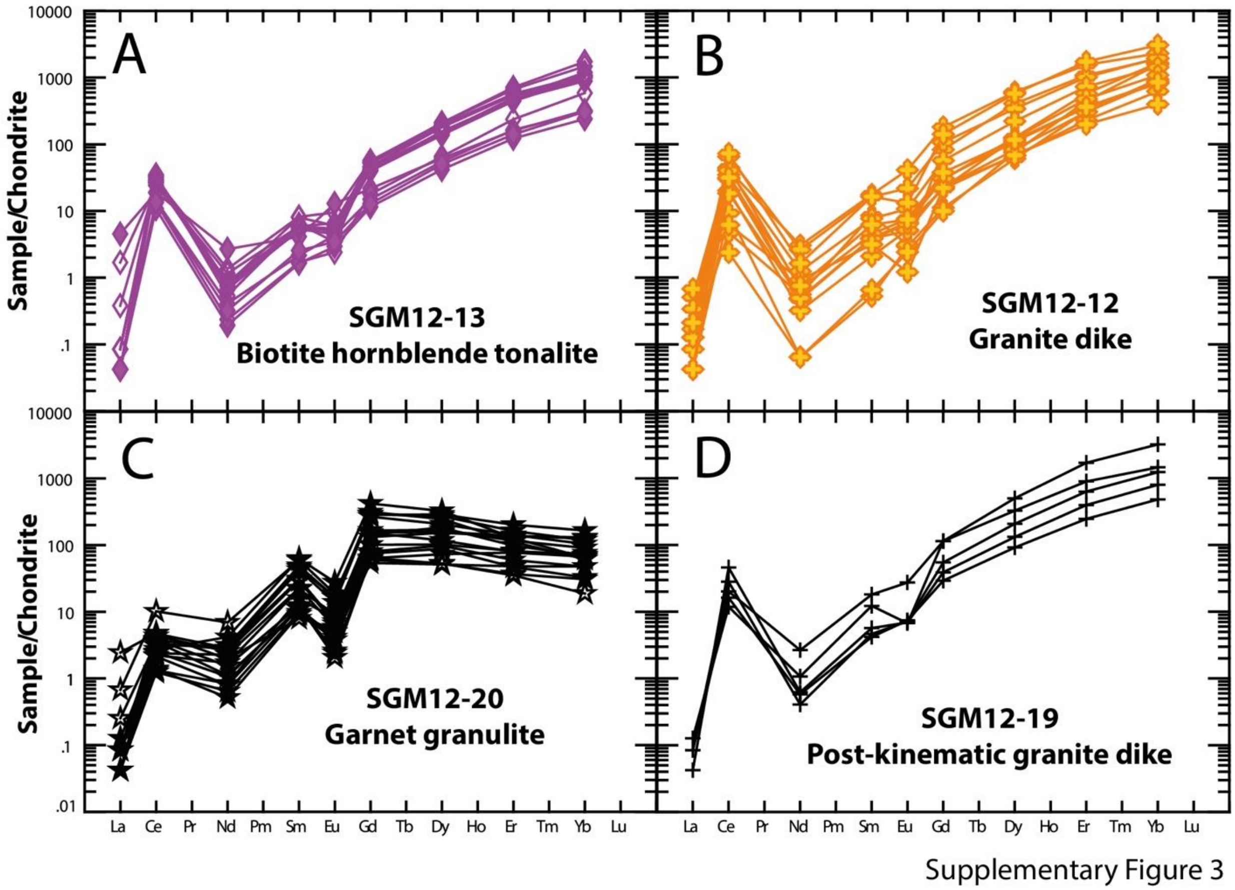

Supplementary Figure 3. Selected chondrite-normalized REE element plots for selected zircons shown in Supplementary Figure 2. Zircons from lower crustal garnet granulite in the Cucamonga terrane (Supplementary Fig. 3C) show depletions in heavy rare earth elements indicating growth with garnet at ca. 86-83 Ma.

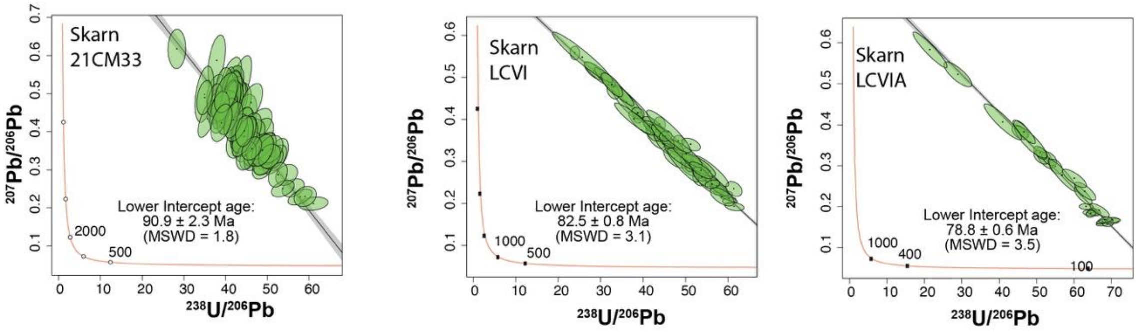

Supplementary Figure 4

Supplementary Figure 4. Tera-Wasserburg diagrams for metamorphic titanite-bearing samples dated in this study. Dates used in calculations are shown by green error ellipses, and rejected samples shown in grey. Error ellipses show 2σ total uncertainty for individual spot analyses, isochrons are indicated by black line with grey error field. Lower intercepts of isochrons give the timing of metamorphic titanite formation/recrystallization. MSWD = Mean square of weighted deviates.
