# Supplementary material for: Magmatic surge requires two-stage model for the Laramide orogeny
Source: Nat Commun. 2023 Jun 29;14:3841. doi: 10.1038/s41467-023-39473-7 (PMC10310782; doi:10.1038/s41467-023-39473-7)
Supplement: Supplementary file 3 — Description of Additional Supplementary Files [file 41467_2023_39473_MOESM3_ESM.pdf]

Description of Additional Supplementary Files

Supplementary Dataset 1. Summary of new LA-SF-ICP-MS and SHRIMP-RG zircon and titanite geochronology in the SCB.

Supplementary Dataset 2. LA-SF-ICPMS zircon U-Pb isotope data.

Supplementary Dataset 3. U-Pb zircon isotope data (SHRIMP-RG).

Supplementary Dataset 4. Zircon trace-element data (SHRIMP-RG).

Supplementary Dataset 5. Zircon trace-element data (LA-SF-ICPMS).

Supplementary Dataset 6. Titanite isotope and trace-element data (LA-SF-ICPMS).

Supplementary Dataset 7. Quartz-garnet oxygen isotope thermometry.

Supplementary Dataset 8. Compilation of U-Pb dates for plutonic rocks in the SCB.

Supplementary Dataset 9. Compilation of major-element geochemistry for plutonic rocks in the SCB, SNB and Cordilleran Anatectic Belt. Data for the SNB compiled from the NAVDAT database (https://www.navdat.org/).
